# Near-Infrared Transillumination for Macroscopic Functional Imaging of Animal Bodies

**DOI:** 10.3390/biology12111362

**Published:** 2023-10-24

**Authors:** Koichi Shimizu

**Affiliations:** 1 School of Optoelectronic Engineering, Xidian University, Xi’an 710071, China; k.shimizu15@kurenai.waseda.jp; 2 IPS Research Center, Waseda University, Kitakyushu 808-0135, Japan

**Keywords:** biomedical imaging, diffusion, functional imaging, medical imaging, near-infrared light, near-axis scattered light, noninvasive measurement, scattering, transillumination imaging

## Abstract

**Simple Summary:**

Advancements in optical technology have revitalized transillumination imaging for biomedical research. Using near-infrared (NIR) light, we have visualized internal structures in animals, even under conditions of heavy optical scattering. By extending the Beer–Lambert law’s applicability through differentiation principles, we can observe real-time physiological changes noninvasively. A hurdle in transillumination is image blurring caused by scattering in body tissues. We have addressed this difficulty by extracting near-axis scattered components from diffuse light, and by employing software techniques such as PSF deconvolution and deep learning. This shift has enabled the clear 3D imaging of internal structures from blurred 2D images. Validation through experimentation with human and animal subjects has underscored the effectiveness of these techniques. Integrating transillumination with modern technology holds great promise for future biomedical applications.

**Abstract:**

The classical transillumination technique has been revitalized through recent advancements in optical technology, enhancing its applicability in the realm of biomedical research. With a new perspective on near-axis scattered light, we have harnessed near-infrared (NIR) light to visualize intricate internal light-absorbing structures within animal bodies. By leveraging the principle of differentiation, we have extended the applicability of the Beer–Lambert law even in cases of scattering-dominant media, such as animal body tissues. This approach facilitates the visualization of dynamic physiological changes occurring within animal bodies, thereby enabling noninvasive, real-time imaging of macroscopic functionality in vivo. An important challenge inherent to transillumination imaging lies in the image blur caused by pronounced light scattering within body tissues. By extracting near-axis scattered components from the predominant diffusely scattered light, we have achieved cross-sectional imaging of animal bodies. Furthermore, we have introduced software-based techniques encompassing deconvolution using the point spread function and the application of deep learning principles to counteract the scattering effect. Finally, transillumination imaging has been elevated from two-dimensional to three-dimensional imaging. The effectiveness and applicability of these proposed techniques have been validated through comprehensive simulations and experiments involving human and animal subjects. As demonstrated through these studies, transillumination imaging coupled with emerging technologies offers a promising avenue for future biomedical applications.

## 1. Introduction

Imaging within the realm of biomedical applications can be categorized into two domains based on object size: macroscopic and microscopic imaging [1,2,3,4,5]. The substantive importance of macroscopic imaging has been demonstrated prominently in medical practices, encompassing X-ray imaging, positron emission tomography (PET), magnetic resonance imaging (MRI), and ultrasonic echo imaging [6,7,8,9,10,11,12]. Although these modalities offer undeniable utility, they are not devoid of limitations. Even with recent progress in X-ray detection [13,14], the ionizing radiation inherent to X-ray imaging engenders challenges related to repeated exposure. Similarly, the utilization of PET and MRI is impeded by the considerable scale of the necessary apparatus, thereby hindering seamless bedside deployment. The domain of ultrasound imaging presents difficulty involving a tradeoff between spatial resolution and penetration depth in animal bodies. An additional contender for noninvasive macroscopic structural imaging of animal bodies has emerged: optical imaging [15,16,17,18,19,20].

The methodology of transillumination imaging, wherein light is projected through one side of an animal body to yield an observable image on the opposite side, has been established [21,22,23,24,25]. This approach, which has been demonstrated historically [26], has not been widely used for the macroscopic imaging of internal body structures in medical practice. This decidedly narrow adoption can be attributed, in part, to the absence of suitable instrumentation for its application. For instance, it has been difficult to use high-power light sources because of the heat dissipated in animal bodies. Furthermore, the sensitivity of image sensors proved insufficient to capture the subtle variations present in transillumination images faithfully. Moreover, the quality of transillumination images notably deteriorated when probing deeper anatomical structures. Recently, a technique was reported that can make animal bodies transparent and which can selectively label the target molecules [27,28,29,30,31,32]. Although this technique is useful for macroscopic organ imaging, it is not applicable to the real-time imaging of live animals.

The impediments outlined above can be surmounted through contemporary optical technologies [33]. The proliferation of high-intensity light sources operating within a narrow wavelength spectrum, along with the emergence of sensitive image-capturing devices, is currently available. Specifically, for the near-infrared (NIR) wavelength range, we possess lasers that exclude heat-producing wavelengths in water, as well as highly sensitive light detectors developed for optical communication purposes [34,35,36,37,38]. Additionally, progress in data processing techniques, notably demonstrated by deep learning, hold the potential to overcome these challenges [39,40,41,42,43,44,45,46,47,48].

In the spectrum spanning from ultraviolet to infrared light, NIR light with a 700–1200 nm wavelength exhibits reduced attenuation when passing through animal bodies [15,16,17,18,19,20]. Leveraging this characteristic along with the background described previously, we have developed various techniques to innovate transillumination imaging. This paper elucidates some of these techniques devised to realize the noninvasive macroscopic and functionally informative imaging of animal bodies within the context of biomedical applications.

## 2. Two-Dimensional Transillumination Imaging

**Figure 1:**
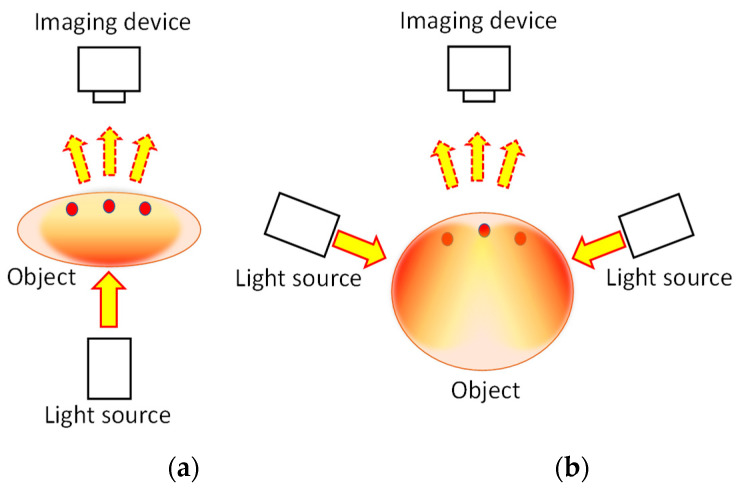
Principle of transillumination imaging: (**a**) transmission mode and (**b**) backscattering mode.

Figure 1 presents the fundamental principle of transillumination imaging [21,22,23,24,25,26,49]. We illuminate the animal body from one side and capture the resulting image from the opposite side (Figure 1a). This technique provides a simple and safe tool to investigate the internal structure of an animal body noninvasively. However, for NIR light, an animal body is regarded as a scattering-dominant turbid medium containing an inhomogeneous, light-absorbing internal structure. The scattering coefficient (*μ*_s_) of human body tissue is typically of approximately 10 mm^−1^ in NIR wavelengths [15,16,17,18,19,20]. This fact implies that the non-scattered component of light decays rapidly within a few millimeters in body tissue. However, when the body is less than a few centimeters thick, the internal light-absorbing structure can be visualized through transillumination imaging [49]. For instance, in human and animal bodies, we can observe a subcutaneous blood vessel network because of the marked differences in the absorption coefficients (*μ*_a_) of hemoglobin and the surrounding tissues [15,16,17,18,19,20].

A transillumination image represents the spatial distribution of light that reaches an imaging device through collimation optics, such as a human eye or a camera. In the transmitted light emerging from an animal body, the image-composing light is not the non-scattered component but the component that results from repeated scattering in the forward direction. When we illuminate an animal body with a collimated light beam, we can observe a portion of the light in the same direction as the incident beam even through a few centimeters of thickness. Animal body tissue is known to have an anisotropy factor (g) close to 1 [15,16,17,18,19,20], which indicates that the scattering pattern of body tissue is highly oriented towards the forward direction. Consequently, some of the light can emerge through the body tissue in the same direction as the incident light after undergoing repeated forward scattering. We refer to this component as near-axis scattered light (NASL) [50]. It shares a similar concept to the snake photon [51,52,53] or quasi-ballistic light [54,55,56,57]. From experimentation, we have confirmed that the NASL intensity remains detectable even after passing through 1 cm thick animal tissue [50]. Because NASL emerges from the animal body in the same direction as the incident light beam, the internal structure can be visualized by employing collimated optics using a regular camera lens with a small aperture.

**Figure 2:**
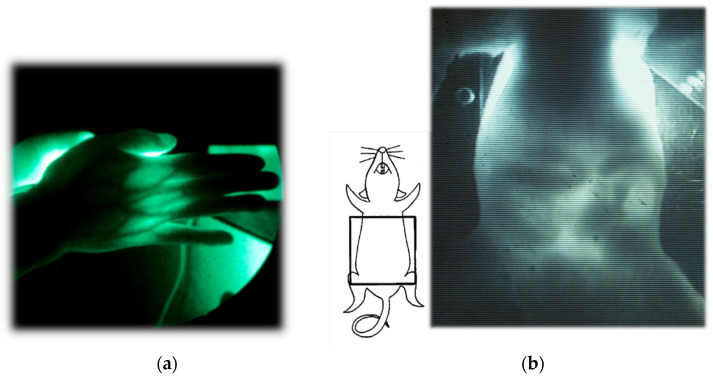
Examples of NIR transillumination images: (**a**) human hand and (**b**) mouse abdomen. Modified from Ref. [49].

Figure 2 presents examples of transillumination images of a human and a mouse [49]. Details of the experiments are reported elsewhere [58]. It is noteworthy that this technique can provide real-time video images with simple instrumentation in safe settings, enabling repeated or continuous bedside monitoring. In the video image of the mouse abdomen, the peristaltic movement of its digestive tract during regular food consumption can be visualized, obviating the need for any unnatural contrast medium required for X-ray fluoroscopy.

In cases where the object body is too thick to allow for light transmission in the incident beam direction, indirect light illumination can be used. Figure 1b presents this illumination technique. Given the highly diffusive nature of general body tissue, characterized by a reduced scattering coefficient *μ*_s_′= ~1 mm^−1^ in NIR wavelengths, the illuminated light from the side propagates almost uniformly after several millimeters of propagation. Consequently, the illuminated light is dispersed uniformly within the body, serving as back-illumination for imaging subsurface absorbing structures. This illumination technique has found application in finger-vein personal authentication [59,60]. Additionally, its application in the daily management of arterio-venous fistulas in dialysis treatment has been reported [61].

## 3. Functional Imaging

One important merit of NIR transillumination imaging is its capability for the noninvasive visualization of physiological changes occurring within animal bodies from an external perspective. Along with the recent progress in optoelectronic devices [62,63], we can leverage the extensive utilization of spectroscopy in the biomedical realm. As explained in detail in Section 2, a transillumination image represents the spatial distribution of NASL observed through collimation optics. The intensity is a consequence of attenuation through animal body tissue, which is a scattering-dominant turbid medium. If we disregard rigor, then the received intensity can be expressed using the following equation.

(1) $$\ln\frac{I_{in}\left(x,y\right)}{I_{out}\left(x,y\right)}=\left[\mu_{s}\left(x,y\right)+\mu_{a}\left(x,y\right)\right]d_{t}\left(x,y\right)-S\left(x,y\right)$$

In this equation, *I*_in_, *I*_out_, *μ*_s_, *μ*_a_, *d*_t_, and *S*, respectively, denote the incident light intensity, output light intensity (i.e., the transillumination image), scattering coefficient, absorption coefficient, path length in body tissue, and increment factor that represents scattering from off-axis directions. The parameters (*x*, *y*) signify the spatial distribution in the plane perpendicular to the optical axis in the z-direction of the Cartesian coordinate system. If we ignore the terms *μ*_s_ and *S*, then this equation reduces to the conventional Beer–Lambert law. Because we cannot neglect these terms with animal body tissue, the Beer–Lambert law is typically deemed inapplicable.

However, by obtaining transillumination images under two different conditions, wherein only the absorption condition varies while the other conditions are kept constant, absorption changes in a linear manifestation of the Beer–Lambert law can be visualized as

(2) $$\ln\frac{I_{2}\left(x,y\right)}{I_{1}\left(x,y\right)}=\left[\mu_{a1}\left(x,y\right)-\mu_{a2}\left(x,y\right)\right]d_{t}\left(x,y\right),$$

where designations 1 and 2, respectively, denote conditions before and after the change. Equation (2) elucidates that by calculating the logarithm of intensity ratios for two images, pre-change and post-change, the resulting image delineates the spatial distribution of absorption changes while mitigating the scattering effect. Because *μ*_a_ is proportional to the molar absorption coefficient *ε* and the absorber concentration *C*, the observed absorption changes typically reflect alterations in either *ε* or *C*. By capturing transillumination images *I*_1_ and *I*_2_ before and after physiological changes within animal bodies, transillumination imaging can capture the internal functional changes manifesting as variations in *ε* or *C*.

**Figure 3:**
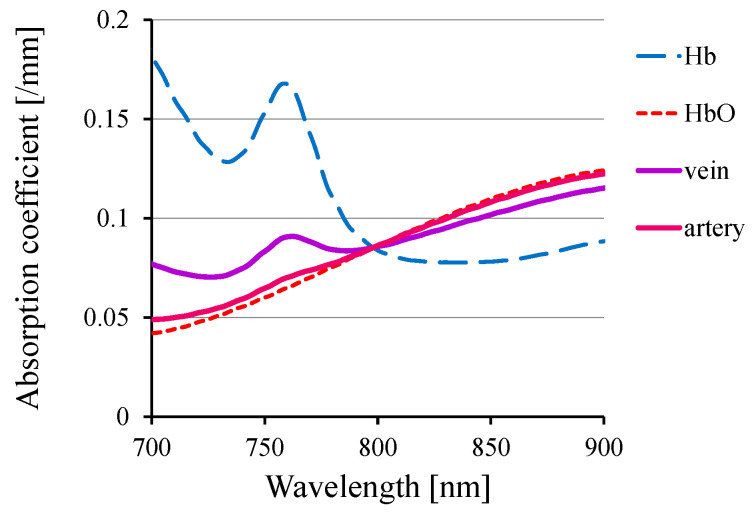
Absorption spectra of hemoglobin and blood.

**Figure 4:**
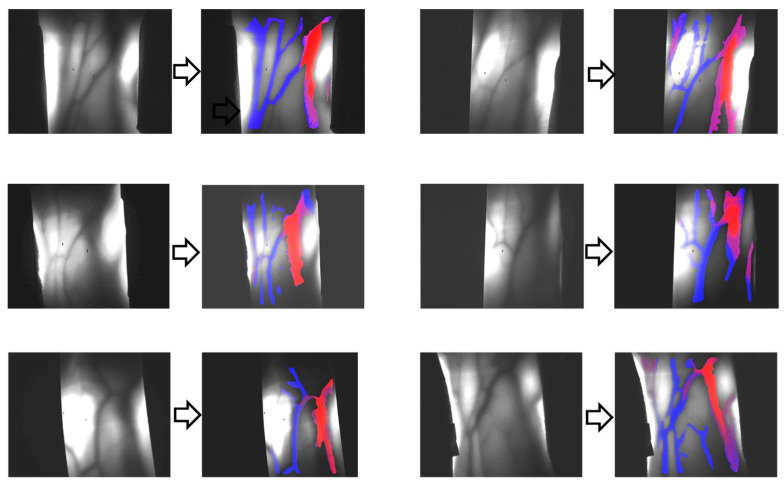
Differentiation of veins (blue color) and arteries (red color) in transillumination images of human adult wrist areas.

One such functional change within animal bodies is the alteration in hemoglobin oxygenation. Figure 3 portrays the absorption spectra of hemoglobin and typical arterial and venous blood, showcasing distinct wavelength-dependent variations in the NIR range. Using these variations, one can visualize the distributions of venous and arterial blood separately. Figure 4 presents the results of venous–arterial differentiation in transillumination images obtained from different human wrists [64].

**Figure 5:**
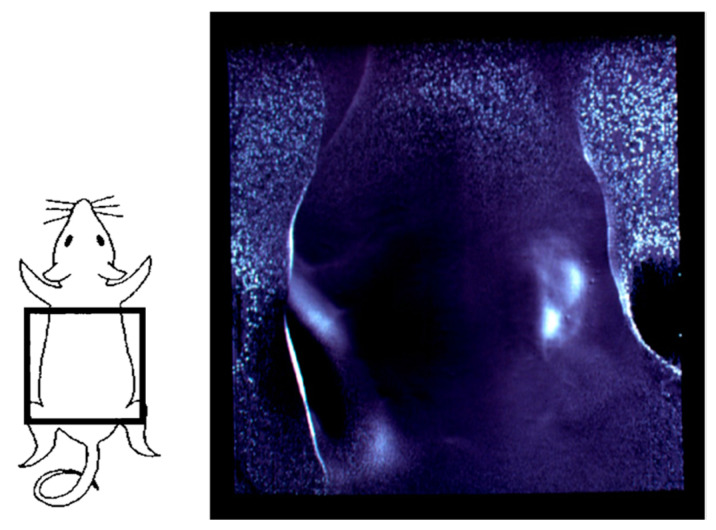
Visualization of blood volume change caused by constricting renal circulation in right kidney. Modified from Ref. [58].

In the isosbestic wavelength, oxyhemoglobin and deoxyhemoglobin exhibit equal absorption. Therefore, by using this specific wavelength in the NIR range (typically of approximately 800 nm for human and rodent blood), one can independently visualize alterations in blood volume irrespective of its oxygenation status. Figure 5 displays an example of the functional imaging outcome, achieved through transillumination images captured before and after a physiological change. In this instance, the suppression of renal circulation because of constricted blood vessels was shown for one of the paired mouse kidneys. This result highlighted the alterations in blood volume within the kidney [58,65].

**Figure 6:**
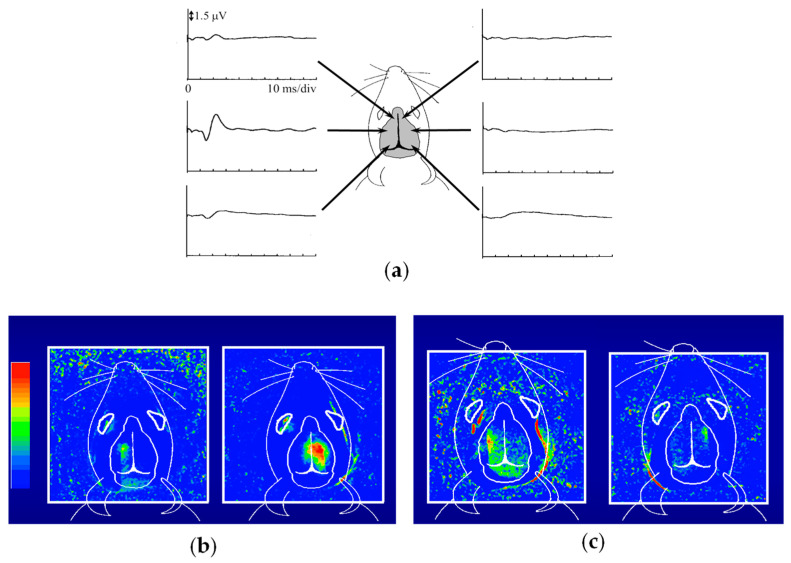
NIR functional transillumination imaging of rat brain: (**a**) EEG identification of activation area for somatosensory stimulation of right forelimb, (**b**) distribution of blood volume change in right/left forearm stimulation, and (**c**) right/left whisker root stimulation.

Figure 6 presents the outcomes of functional imaging in a rat brain [65]. Electro-stimulation was applied to one of the rat’s forelimbs or whisker roots, with the intention of visualizing the corresponding local changes in its brain. Before conducting optical imaging, we identified the cerebral cortex responsible for somatosensory perception. Figure 6a shows the electroencephalograms (EEGs) observed via metal electrodes in direct contact with the mouse head during right forelimb stimulation. Figure 6b presents the functional imaging results, obtained from the transillumination images taken before and after forelimb stimulation. The localized increase in light attenuation, brought about by an augmented blood volume, agreed well with the EEG-identified regions. Stimulation of the right forelimb yielded an activated region in the left cerebral hemisphere. Upon switching the stimulation from the right to the left forelimb, the activated area transitioned symmetrically across the longitudinal cerebral fissure that separates the right and left cerebrum hemispheres. As depicted in Figure 6c, stimulation of the right/left whisker root caused different activated patterns compared to the forelimb stimulation, but remained within the respective left and right hemispheres. These positions and the localized dispersion in the somatosensory area closely correlated with measurements obtained through microelectrodes inserted into the brain [66,67]. It is noteworthy that these observations were noninvasive, requiring no incision or skull opening. The sole intervention involved shaving the hair on the rat’s head to enhance the clarity of the transillumination images.

## 4. Hardware-Based Scattering Suppression

**Figure 7:**
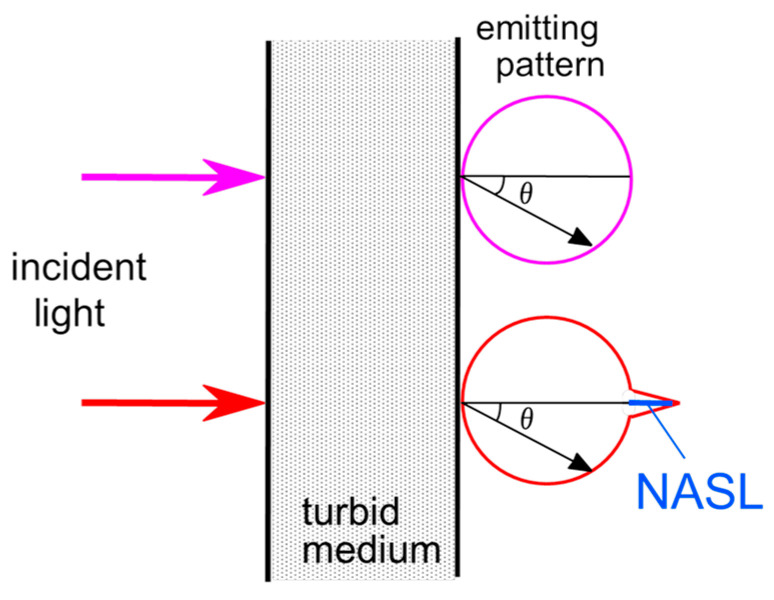
Principle of selective detection of near-axis scattered light (NASL) component from predominant diffuse scattered light: Light distribution of diffuse component only (pink color) and that with NASL component (red color). The NASL component magnitude is exaggerated for illustrative purposes, emphasizing the underlying concept.

As elaborated in the preceding chapter, we can improve transillumination imaging by suppressing the strong scattering effects that occur in body tissue. By extracting the NASL component through effective elimination of the dominant diffuse light component, we not only improve transillumination images but also enable computed tomography (CT) using the directly propagated light component [68,69]. Numerous techniques using coherency, polarization, and time-of-flight of the probing light have been reported for extracting the non-scattered component [70,71,72,73]. However, because NASL results from repeated forward scattering, these techniques struggle to distinguish NASL from the dominant diffuse light. Therefore, we specifically examined the extraction of the NASL component from the diffuse light. Figure 7 presents details of the principle underpinning our developed NASL extraction method for scattering suppression [50]. Typically, the reduced scattering coefficient of animal tissue ranges around 1 mm^−1^ in NIR wavelength [15,16,17,18,19,20]. Consequently, the angular distribution of light output from a diffuse medium becomes nearly uniform after traversing body tissue exceeding 1 cm in thickness. Yet, as outlined in Section 2, we anticipate an NASL component output from animal tissue with detectable intensity. Despite its intensity being notably smaller than the dominant diffuse component, we can extract it using the following differential approach [50].

When we measure output light from the body surface using a finely collimated detector, the angular distribution of the diffused component is articulated as

*I*_out_(*θ*) = C_o_ *I*_in_ cos*θ*, (3)

where *I*_out_, *I*_in_, *θ* (≠0), and C_o_, respectively, stand for the output and incident light intensity, emission angle, and a proportional constant. In the on-axis direction of the incident collimated light beam, the received intensity is as follows.

*I*_out_(0) = C_o_ *I*_in_ + *I*_NASL_ (4)

Here, *I*_NASL_ denotes the NASL intensity. Consequently, we can extract the NASL component by measuring the light intensity at two angles, *θ* = 0 and *θ*_m_, using the equation

*I*_NASL_ = *I*_out_(0) − *I*_out_(*θ*_m_)/cos*θ*_m_, (5)

where the measurement angle *θ*_m_ ≠ 0.

**Figure 8:**
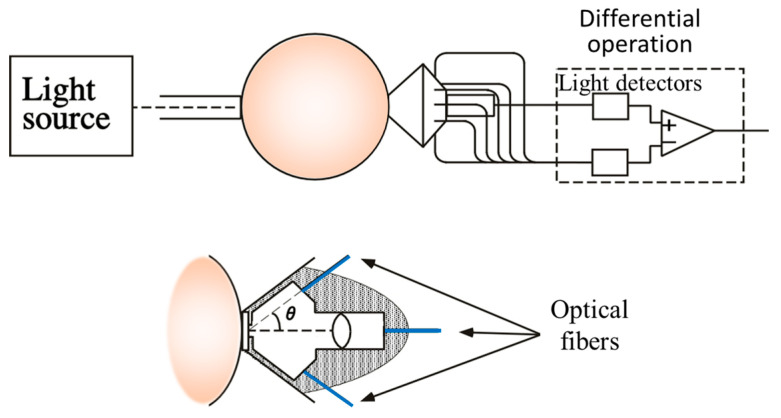
Outline of detector system used to measure NASL intensity from on-axis and off-axis scattered light through the animal body. Modified from Ref. [50].

**Figure 9:**
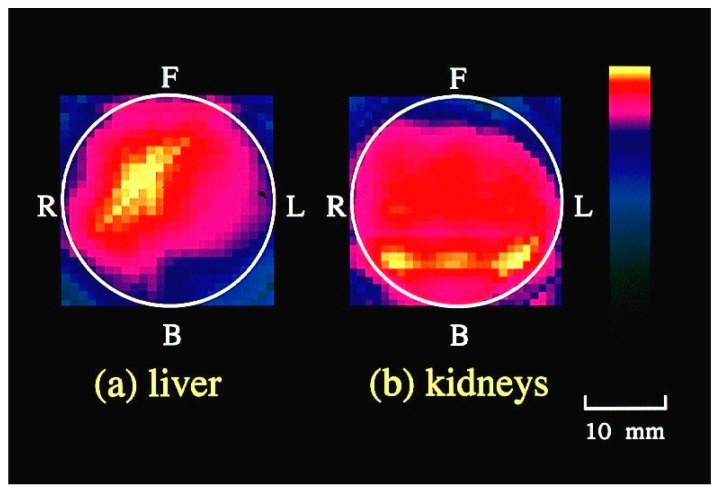
Cross-sectional images of mouse abdomen reconstructed from NASL components. F, B, R and L respectively represent front, back, right and left around the body axis. Modified from Ref. [50].

To implement this principle, we designed a light detector, as depicted in Figure 8 [50]. To mitigate the diffuse reflection on the body surface, optical fibers in contact with the body surface were employed. The fiber holder on the body surface secured on-axis and off-axis fibers at angles of 0 and 40 degrees, respectively. The outcomes of CT imaging on a mouse abdomen using the developed detector are presented in Figure 9. Cross-sectional images obtained at different heights along the mouse body axis successfully revealed blood-rich organs such as the liver and kidneys [50]. We have developed other hardware-based techniques to suppress scattering effects, and have subsequently confirmed their efficacy through experimentation [74,75].

## 5. Software-Based Scattering Suppression

**Figure 10:**
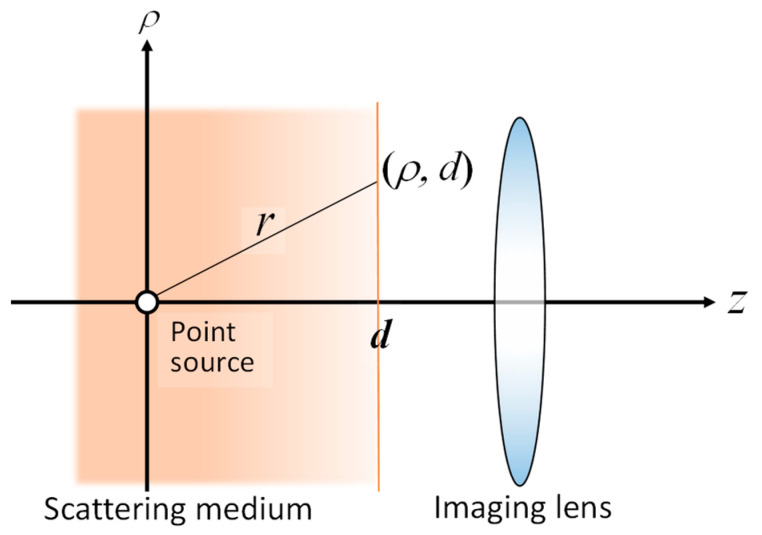
Geometrical model of point-spread function (PSF).

The blurring of transillumination images can be modeled as the convolution of the blur-less image with a point spread function (PSF) of the blur. Consequently, if we have a PSF, we can restore the blur-less image by deconvolving the blurred image with the PSF. By applying diffusion approximation to the radiative transfer equation, we derived the theoretical PSF for the model shown in Figure 10 [76].

(6) $$PDF\left(\rho \right)=C\left[\left(\mu_{s}{}^{\prime }+\mu_{a}\right)+\left(\kappa_{d}+\frac{1}{\sqrt{\rho^{2}+d^{2}}}\right)\frac{d}{\sqrt{\rho^{2}+d^{2}}}\right]\frac{\exp\left(-\kappa_{d}\sqrt{\rho^{2}+d^{2}}\right)}{\sqrt{\rho^{2}+d^{2}}}$$

Therein, *κ*_d_ = [3*µ*_a_(*µ*_s_′ + *µ*_a_)]^1/2^; the radial distance *ρ* = (*x*^2^ + *y*^2^)^1/2^. In this equation, C, *µ*_s_′, *µ*_a_, and *d*, respectively, represent the constant with respect to *ρ* and *d*, the reduced scattering coefficient, the absorption coefficient, and the absorber depth.

**Figure 11:**
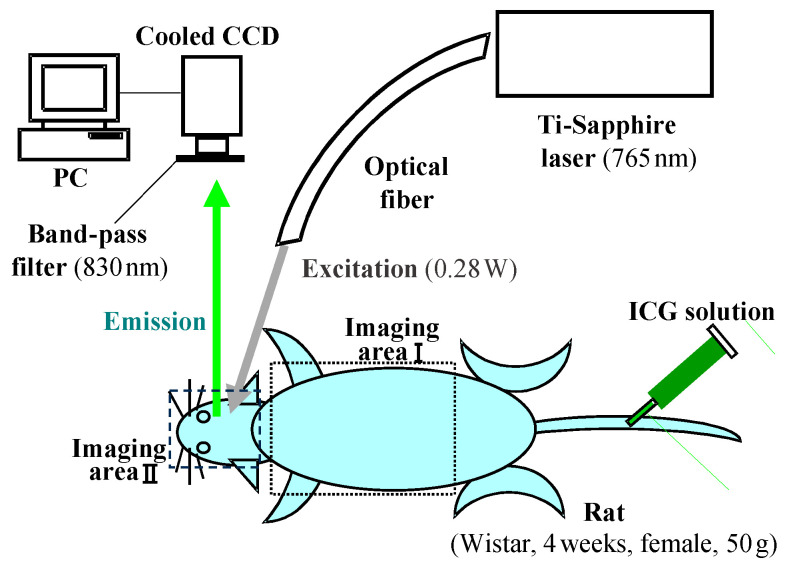
Transcutaneous fluorescent imaging of rat to verify applicability of the PSF deconvolution technique. Modified from Ref. [76].

**Figure 12:**
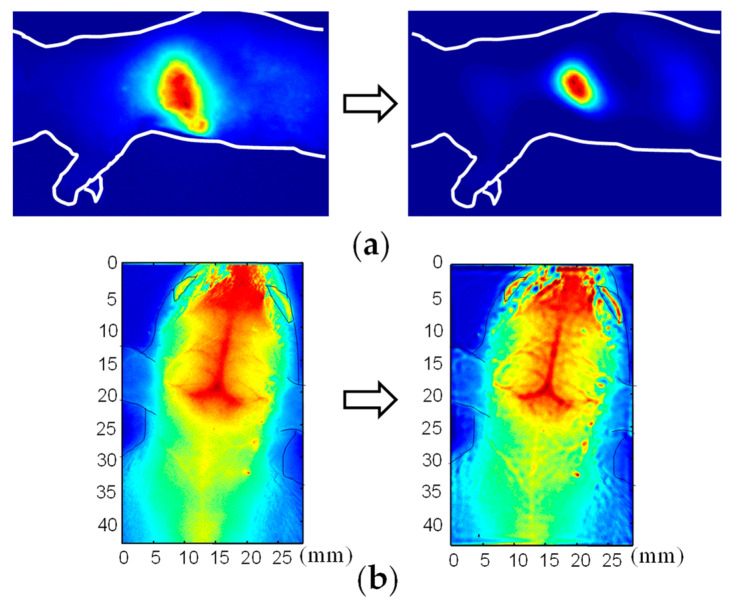
Results of PSF deconvolution: heart (**a**) and fine blood vessels (**b**) became identifiable. Modified from Ref. [76].

The validity and applicability of the derived PSF were confirmed through experimentation. Figure 11 and Figure 12, respectively, present an outline of the imaging experiments and the results obtained before and after deconvolution [76]. Although the PSF was designed initially for a light source in a turbid medium, it can be extended to a transillumination image. A transillumination image is commonly a distribution of light-absorbing structures in the medium. Nevertheless, if the structure is considered as an ensemble of light-absorbing or light-absent points, then one can employ the same equation (Equation (6)) as the PSF for the transillumination image [77].

**Figure 13:**
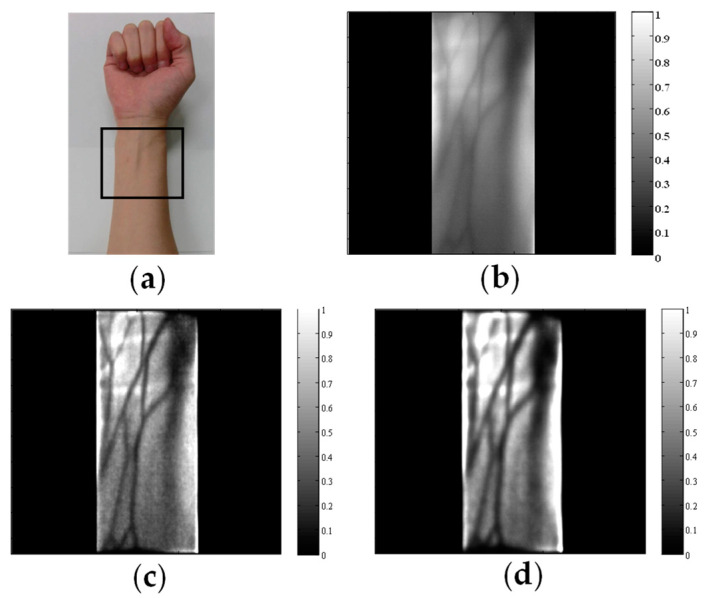
Results of PSF deconvolution: (**a**) visible image and ROI, (**b**) NIR transillumination image, (**c**) deconvolution with the PSF of 2.1 mm depth, and (**d**) deconvolution with the PSF of 4.0 mm depth.

The PSF of Equation (6) is formulated as a function of the absorber depth *d*. Consequently, appropriate scattering suppression is applied to a specified depth. Figure 13 portrays the difference between adult arm images acquired through deconvolution with different depth parameters. With small *d* values in the PSF, shallow veins were deblurred clearly. With large *d* values in the PSF, deep veins and arteries were deblurred appropriately [78].

Another software-based scattering suppression method uses the deep learning principle. When observing a blurred image through a turbid medium, the observer’s mind effectively envisions the unblurred image as if the medium were clear. This cognitive process, which has typically been honed through experience or training, can be implemented within a deep neural network [79]. In a computational framework, a neural network is trained using numerous pairs of training data, consisting of sets of blur-less images and their corresponding blurred counterparts. After extensive training, this network can output blur-less images from previously unseen blurred inputs. Although accumulating a sufficient number of training pairs of transillumination images might prove challenging with specific biomedical applications, the PSF convolution method described above enables the generation of numerous blurred images. We applied this technique to blurred transillumination images of subcutaneous blood vessel networks.

**Figure 14:**
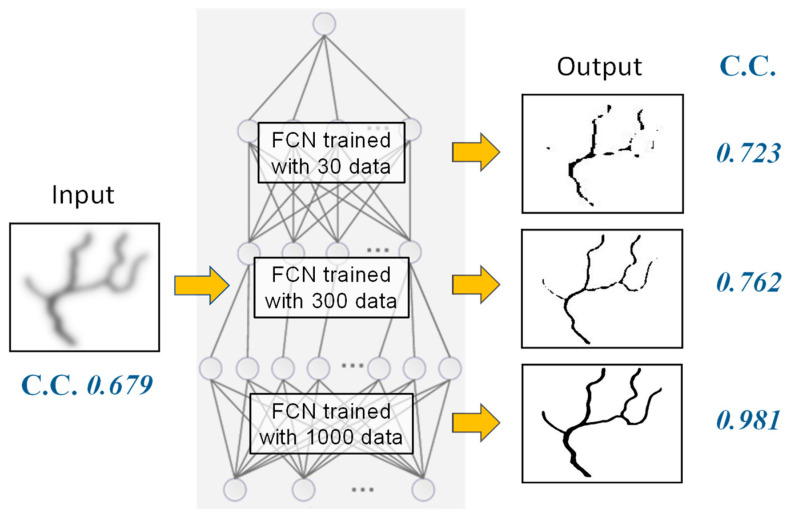
Dependence of output image quality on the number of training data in scattering suppression with FCN. C.C.: correlation coefficient to blur-less (ground truth) image.

To evaluate the feasibility of this approach, we conducted simulations using blood vessel patterns. For the neural network model, we employed a fully convolutional neural network (FCN) based on a U-net architecture with skip connections [80,81,82,83]. Our training dataset comprised blur-less blood vessel images and blurred images generated using the PSF of Equation (6) at varying depths *d*. By altering the orientation and depth, we generated multiple images from a single original blood vessel image. Figure 14 depicts the dependence of scattering suppression efficacy on the number of training data. The correlation coefficient between the blur-less (ground truth) image and the output image was improved with a sufficient number of training data. Following extensive training with diverse data, the trained FCN effectively outputs clear blur-less images for blurred inputs obtained at different absorber depths, all without requiring depth information.

## 6. 3D Transillumination Imaging

In the realm of biomedical applications, the significance of three-dimensional (3D) imaging for assessing the macroscopic internal structure of animal bodies has been exemplified in X-ray CT, MRI, and ultrasound echo imaging. As shown in Section 2, transillumination images inherently remain two-dimensional (2D). During the process of scattering suppression, we recognized that depth information resides within a blurred 2D image. According to the PSF of Equation (6), the extent of blurring and the depth of the absorbing structure share a one-to-one correspondence. In other words, one can deduce the depth from the blurriness present in a single 2D transillumination image.

**Figure 15:**
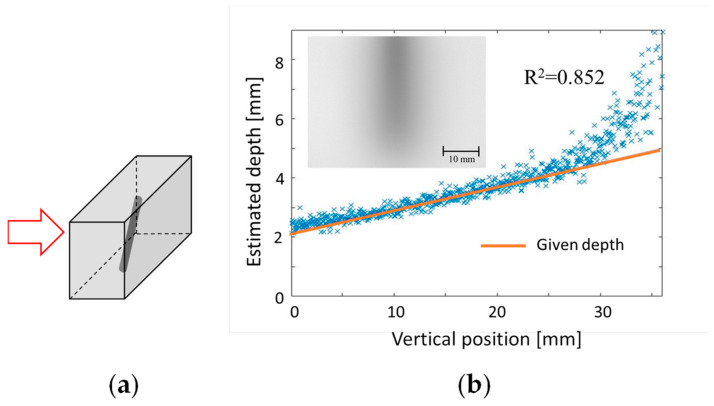
Depth estimation of curvilinear structure in turbid medium: (**a**) light-absorbing bar in tissue-simulating medium, and (**b**) depth estimation from a blurred transillumination image. Modified from Ref. [49].

Building on this concept, we have developed two techniques for reconstructing 3D structures from a single-shot transillumination image [49]. One approach targets curvilinear structures such as subcutaneous blood vessels. The other approach specifically examines solid objects, such as cancer lesions within normal tissue. For the first technique, we established a database that provides absorber depths based on inputs such as the spread and contrast of the blurred image. The transillumination image of the curvilinear structure was partitioned into smaller segments. The depth of each segment was then estimated using the database. The validity of this technique was confirmed through experimentation using an absorber at a known depth in a turbid medium simulating human body tissue. Figure 15 presents an example of depth estimation results. A light-absorbing straight stick was placed at an angle in a tissue-simulating turbid medium, as shown in Figure 15a. Using the proposed technique, we were able to estimate the linear variation of the depth, reaching approximately 25 mm, from a single transillumination image, shown in Figure 15b [49].

**Figure 16:**
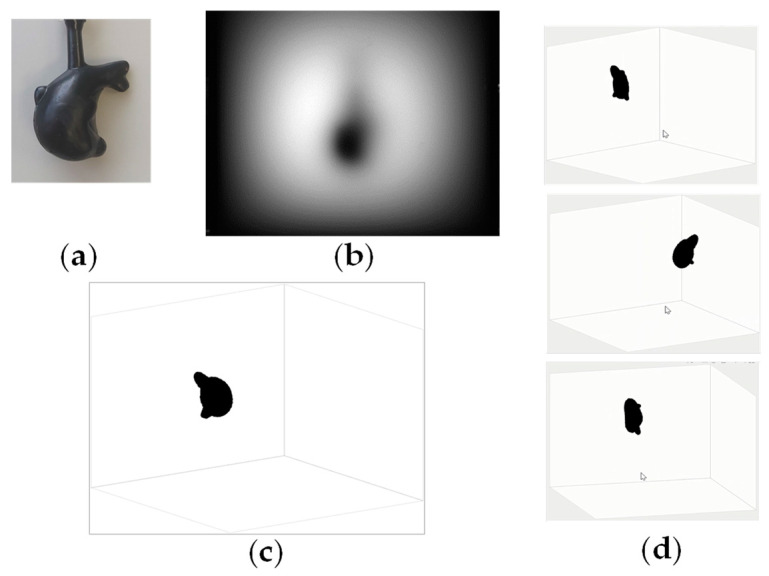
Three-dimensional imaging of a solid object using focus stacking principle: (**a**) object appearance, (**b**) transillumination image in turbid medium, (**c**) result of 3D reconstruction, and (**d**) reconstructed object viewed from different angles. Modified from Ref. [49].

The second technique leverages the focus-stacking principle [84,85,86,87,88]. Using the deconvolution operation on a blurred transillumination image with a depth-dependent PSF (such as Equation (6)), only the image portion at a specific depth *d* is focused correctly. Through iterative deconvolution at varying depths, we acquire multiple images with different in-focus regions of known depths. Using these images, one can compile a composite image in which all segments are in focus and all are annotated with their respective depths. By concurrently applying scattering suppression and depth mapping, it is possible to reconstruct a deblurred 3D image of absorbing structures in a turbid medium. The effectiveness of this technique was examined through experimentation. Figure 16 depicts the object’s appearance, its transillumination image in a tissue-simulating turbid medium, and the resultant 3D reconstruction. These analyses confirm the feasibility of obtaining 3D internal structures from a single blurred transillumination image [49].

**Figure 17:**
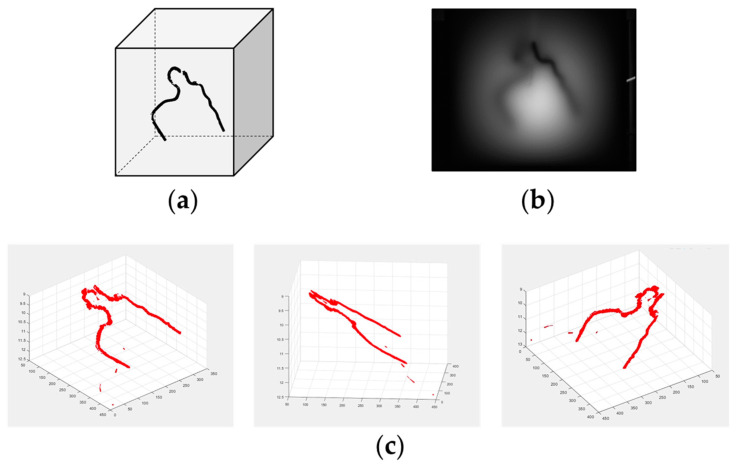
Clear 3D imaging from single transillumination image using FCN and CNN: (**a**) object structure, (**b**) transillumination image in turbid medium, and (**c**) 3D images viewed from different angles. Modified from Ref. [79].

To enhance the utilization of the one-to-one correspondence between blurriness and depth, we incorporated the principles of deep learning into this task. When observing objects at different depths in murky water, the degree of blurring is contingent on the depth. By repeatedly experiencing this phenomenon while having knowledge of the object depths, one can learn to estimate depth from the degree of blur. Therefore, by training a neural network on a computer with numerous training pairs of blurred images and their corresponding depths, a neural network capable of outputting depth for a given blurred image input can be developed. The feasibility of this concept was verified through experimentation. Figure 17 portrays the object structure, its 2D transillumination image through a tissue-simulating medium, and the resultant 3D reconstruction. The deep learning network (FCN) explained in Section 5 eliminated image blur, whereas the 3D structure was reconstructed using a convolutional neural network (CNN) trained on 60,000 pairs of images generated through convolution with the PSF of Equation (6) [79].

**Figure 18:**
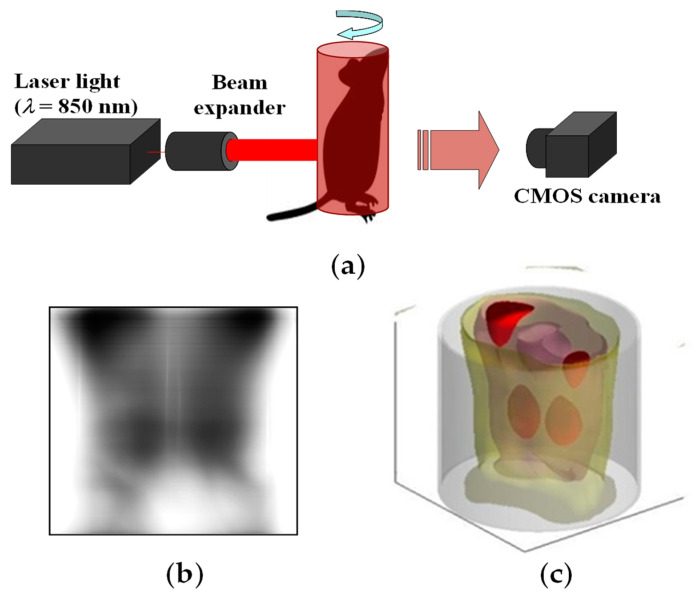
Clear 3D imaging from multiple transillumination images with PSF deconvolution implemented in backpropagation: (**a**) experimental setup, (**b**) transillumination image after deconvolution, and (**c**) reconstructed 3D image of mouse abdomen. Modified from Ref. [77].

The 3D reconstruction from a single-shot transillumination image proves to be particularly valuable in practical scenarios where imaging opportunities are limited to just one attempt. However, this method entails an important limitation in that it cannot restore structures within completely shadowed areas. For situations in which multiple transillumination imaging from various perspectives is feasible, we have developed an alternative technique. For this approach, scattering effects are mitigated through repeated PSF deconvolution combined with the back-projection algorithm in CT reconstruction [77]. The effectiveness of this technique was demonstrated in animal experiments. Figure 18 outlines the experimental setup, presents an example of a transillumination image, and showcases the resulting 3D reconstruction of the absorbing structure within a mouse abdomen. It is noteworthy that blood-rich organs such as kidneys and portions of the liver were visualized successfully in the 3D image [77].

## 7. Conclusions

This review article, written for the Special Issue of “Development of New Optical Techniques and Methods for Basic Biology and Biomedical Applications,” introduces an optical technique that has been greatly enhanced by recent technological advances and which is useful today for biomedical applications.

In the pursuit of imaging the internal macroscopic structure of animal bodies, optical transillumination imaging techniques have been innovated through contemporary technologies. Using NIR light, we can effectively capture light that has traversed animal bodies with thicknesses exceeding several cm. Because of strong light scattering in body tissue, non-scattered light passing through an animal body becomes less than the detectable limit for thicknesses of approximately millimeters. However, we noticed the existence of a light-component NASL, which results from repeated forward scattering and which propagates directly through body tissues with thicknesses of approximately centimeters. Through the use of collimation optics, we can acquire transillumination images of internal light-absorbing structures within animal bodies.

This technique enables the visualization of blood vessel networks in human hands, arms, and feet. For thicker body segments, a side illumination method is applicable. In this method, diffused light in the body tissue acts as backlighting, enabling the capture of transillumination images of subsurface structures. This method is useful for the routine management of arteriovenous fistulas, which are constructed in patients’ bodies for dialysis treatments. In animal experiments, the movement of internal organs can be monitored through real-time video imaging. The peristaltic motion of the digestive tract during regular food consumption can also be observed without the need for an artificial contrast agent.

Transillumination imaging is also extremely useful for the functional imaging of animal physiology. The intensity ratio between transillumination images before and after a functional change yields an image of the physiological change that occurred within the body. By exploiting differences in the absorption spectra between oxyhemoglobin and deoxyhemoglobin, we have differentiated arteries from veins in human wrists. Using the isosbestic wavelength in the NIR range (approximately 800 nm), it is possible to discern changes in blood volume independently of oxygenation variations. Alterations in renal circulation were visualized in mouse kidney images. The cerebral cortex regions activated via somatosensory stimulation were also visualized in transillumination images of mouse brains. Commissural responses between the right and left hemispheres of the cerebrum were visualized in response to forelimb and whisker stimulation.

A key challenge for biomedical transillumination imaging is image blurring caused by strong light scattering in body tissues. This shortcoming can be addressed by using NASL as the image-forming beam. Based on the differential principle, we have devised a technique to extract the subtle NASL component from the predominant diffuse component in the transmitted light through an animal body. To implement this technique, a detector unit was designed, incorporating optical fiber detectors in both on-axis and off-axis configurations. With this unit integrated into our experimental setup, we achieved optical CT imaging of a mouse abdomen.

The effective suppression of scattering effects can also be accomplished using software-based techniques. A PSF describing the blurring caused by scattering was derived theoretically as a function of the depth of a light-emitting point in a turbid medium. The blurring of transcutaneous fluorescent images was mitigated effectively through PSF deconvolution. This method proved to be applicable to the transillumination imaging of absorbing structures, as well. For instance, the blurring of human blood vessels at varying depths was reduced selectively using the depth-dependent PSF for transillumination images of an adult arm.

An alternative software-based approach involves the application of deep learning principles. An FCN was trained on numerous pairs of blur-less and corresponding blurred images. An extensive set of training pairs was generated by convolving the blur-less image with the derived PSF across various absorber depths. When a new blurred transillumination image was input into the trained FCN, it was able to output a deblurred image, even without prior knowledge of the absorber depth.

Finally, we attempted to elevate the transillumination imaging from 2D to 3D. Subsurface structures of curvilinear absorbing objects in turbid media, such as subcutaneous blood vessels, were reconstructed in 3D using a database capable of providing the absorber’s depth for inputting image contrast and blur spread. For solid absorbers, the 3D structure was reconstructed from a single transillumination image using the focus-stacking principle. Using this technique, multiple images were generated by deconvolving the transillumination image using PSFs of varying object depths *d*. From each segment of the transillumination image, we selected the best focused part among the deconvoluted images, and determined the depth *d* used for deconvolution. By assembling the chosen segments in their original positions, we were able to reconstruct an all-in-focus image with depth mapping.

Furthermore, the technique with deep learning principles was extended to 3D reconstruction from a single transillumination image. A CNN was trained on pairs of absorber depths and blurred images generated using PSFs of given absorber depths. After sufficient training, the trained CNN was able to output the correct depth of the absorbing structure for the input of a new blurred image. By combining the blur-less image from the FCN and the depth information from the CNN, we reconstructed 3D internal structures in turbid media. If we can capture transillumination images from different perspectives, then 3D reconstruction is attainable without any concern about invisible shadow areas. By incorporating the PSF deconvolution process into the back-projection algorithm, 3D transillumination images of a mouse abdomen were obtained with no shadowed regions.

In conclusion, optical transillumination imaging, although its future potential is recognized, has not yet found widespread practical application. We have continued to innovate this technique using recent technological advancements. Biological safety and practical convenience are essential features of NIR transillumination imaging. Repeated imaging or continuous real-time monitoring using a compact bedside device is now achievable. Moreover, the costs of manufacturing and of the actual use of such equipment are assumed to be much lower than the costs of conventional medical imaging equipment. Noninvasive transillumination imaging of physiological functions will prove to be a valuable tool for various biomedical applications. Furthermore, this advancement will fundamentally reduce the necessity of sacrificing animals for experimentation that conventionally requires open-body observations.

However, the suppression of scattering effects remains a challenge in enhancing the quality of transillumination images. Undoubtedly, continued efforts to address this issue will ultimately engender important advancements and the widespread adoption of the transillumination imaging methodology.

## Data Availability

Data sharing is not applicable to the study described in this article.
